# Effect of Block Polyether as an Interfacial Dispersant on the Properties of Nanosilica/Natural Rubber Composites

**DOI:** 10.3390/polym17081091

**Published:** 2025-04-17

**Authors:** Ying Liu, Jiahui Mei, Depeng Gong, Yanjun Chen, Chaocan Zhang

**Affiliations:** School of Materials Science and Engineering, Wuhan University of Technology, Wuhan 430070, China; ly2860495025@163.com (Y.L.); m2bd4l@163.com (J.M.); gdp@whut.edu.cn (D.G.); yanjunchen@whut.edu.cn (Y.C.)

**Keywords:** block polyethers, silica, natural rubber, dispersion, surface modification

## Abstract

To enhance the dispersion of silica within a natural rubber (NR) matrix and improve the modification efficiency of the silane coupling agent, a novel interfacial dispersant composed of block polyether with a PEO-PPO-PEO structure is employed in this study. This block polyether, consisting of poly(ethylene oxide) (PEO) and poly(propylene oxide) (PPO), serves to reduce the surface energy of silica and improve its compatibility with the rubber matrix. Three types of block polyethers with different hydrophilic–lipophilic balance (HLB) values of 8, 13, and 22 are selected to regulate the surface tension of silica. Subsequently, bis[γ-(triethoxysilyl)propyl] tetrasulfide (TESPT) is used to further modify the silica surface, aiming to prepare high-performance rubber composites. The results indicate that the HLB value of the block polyether has a significant influence on the system. Compared with block polyethers having HLB values of 8 and 22, the block polyether with an HLB value of 13 demonstrated superior silica dispersion, leading to enhanced filler–rubber interfacial interactions. Consequently, both the mechanical properties and processability of the NR composites were substantially improved. When the dosage of this block polyether was 1 phr, the composite exhibited a tensile strength of 28.9 MPa and an elongation at break of 523%.

## 1. Introduction

Natural rubber (NR) is a naturally occurring polymer compound, primarily composed of cis-1,4-polyisoprene [1,2]. As an elastomer, it exhibits excellent processability, resistance to various media, and favorable chemical and physical properties. As a result, NR is widely applied in diverse fields such as consumer industries, biomedicine, transportation, and engineered products [3,4,5]. In the rubber industry, carbon black is the most commonly used reinforcing filler. Acting as a reinforcing skeleton within the rubber matrix, carbon black particles significantly enhance the strength, abrasion resistance, and weather resistance of rubber products [6,7,8]. With the adjustment of industrial structures and the increasing demand for colored rubber products, nanosilica (SiO_2_) has been extensively adopted, ranking second only to carbon black in terms of usage in rubber reinforcement applications [9]. Compared with carbon black, nanosilica can effectively reduce the hysteresis and rolling resistance of rubber composites, enhance their wet traction performance, and simultaneously impart excellent mechanical properties, electrical insulation, and weather resistance [10,11,12]. Therefore, in response to the growing demand for green, energy-efficient, and high-performance materials, the use of nanosilica to reinforce rubber has become a prominent research focus.

However, the surface of nanosilica contains a large number of silanol groups, resulting in a high cohesive energy density between nanoparticles, which leads to a strong tendency for agglomeration. Moreover, the polar nature of nanosilica leads to poor compatibility with the non-polar natural rubber matrix, making its dispersion difficult and significantly weakening its reinforcing effect [13,14,15]. To address this issue, various interfacial modifiers have been introduced into silica/natural rubber (SiO_2_/NR) composites to enhance the interfacial compatibility between the two phases [16,17]. Among these, the surface modification of silica using silane coupling agents is the most widely adopted approach, with bis[γ-(triethoxysilyl)propyl] tetrasulfide (TESPT) being the most commonly used silane coupling agent [18,19]. During the modification process, the method of applying the coupling agent is also crucial for optimizing the final performance of the rubber materials. Currently, two main modification strategies are employed: the one-step method (OSM), in which in situ surface modification of silica is performed during mechanical mixing, and the two-step method (TSM), which involves the pre-modification of silica in the liquid phase prior to mechanical blending with rubber [20,21].

You et al. [22] prepared SiO_2_/NR composites using the two-step method. TESPT hydrolysate was first added to a silica slurry to obtain surface-modified silica, which was then compounded with natural rubber to fabricate the rubber composites. When the silica loading was 50 phr, the composite exhibited a tensile strength of 12 MPa, an elongation at break of 798%, a modulus of 1.2 MPa at 100% strain, and 3.3 MPa at 300% strain. Pattanawanidchai et al. [23] modified precipitated silica in an aqueous medium via a xanthate esterification reaction and subsequently prepared SiO_2_/NR composites with a silica content of 50 phr. When the zinc salt of dithiocarbamate-modified silica (ZMS) was introduced at a concentration of 12%, the resulting composite exhibited a tensile strength of 15.1 MPa, an elongation at break of 555%, and a modulus of 1.8 MPa at 100% strain. The two-step method not only involves a relatively complex processing procedure, but may also impose an environmental burden. In recent years, the one-step method, characterized by its simplified processing, has attracted considerable attention from researchers. Surya et al. [24] investigated the effects of alkanolamide (ALK) and 3-aminopropyltriethoxysilane (APTES) on the performance of SiO_2_/NR composites with a silica loading of 30 phr. The results indicated that ALK exhibited superior overall performance compared to APTES. When the ALK loading was 5 phr, the composite achieved a tensile strength of 25.5 MPa, an elongation at break of 985%, a modulus of 1.2 MPa at 100% strain, 3.1 MPa at 300% strain, and a Shore A hardness of 49. Xiao et al. [25] prepared high-silica-loaded (70 phr) natural rubber composites by combining polysorbate-20 (TWEEN-20) with bis[γ-(triethoxysilyl)propyl] tetrasulfide (TESPT), with a total modifier content of 7 phr. When the mass ratio of TWEEN-20 to TESPT was 1:1, the resulting composite exhibited a tensile strength of 23 MPa, an elongation at break of 500%, a modulus of 3 MPa at 100% strain, 11.8 MPa at 300% strain, and a Shore A hardness of 70. Ye et al. [26] employed bis(epoxypropyl) polysulfide (BEP) as a modifier for silica and found that the synergistic use of BEP and TESPT significantly improved the dispersion of silica within the solution-polymerized styrene–butadiene rubber (SSBR) matrix. When the BEP:TESPT ratio was 2:5, the composite exhibited a tensile strength of 19.8 MPa, an elongation at break of 422%, a modulus of 2.4 MPa at 100% strain and 12.8 MPa at 300% strain, and a Shore A hardness of 60. Sae-oui et al. [27] investigated the effects of two silane coupling agents, TESPT and 3-thiocyanatopropyltriethoxysilane (Si-264), respectively, on the properties of silica/chloroprene rubber (CR) composites. They reported that when both coupling agents were added at 3 phr, the composites achieved a tensile strength of 26 MPa, an elongation at break of approximately 850%, a modulus of 1.7 MPa at 100% strain, and a Shore A hardness of 62. Xu et al. [28] studied the influence of acetone extract (AE) from natural rubber on the structure and mechanical properties of silica/natural rubber composites. When the AE loading was 4 phr, the composite demonstrated a tensile strength of 21.12 MPa, an elongation at break of 733%, a modulus of 1.1 MPa at 100% strain, 3.0 MPa at 300% strain, and a Shore A hardness of 46.

Due to the highly polar nature and strong tendency to agglomerate, nanosilica is difficult to achieve uniform dispersion within the non-polar natural rubber (NR) matrix. Additionally, the commonly used silane coupling agent, bis[γ-(triethoxysilyl)propyl] tetrasulfide (TESPT), is relatively hydrophobic and tends to distribute preferentially in the rubber phase, which may reduce its surface modification efficiency with the hydroxyl groups on the silica surface. In this study, a novel nonionic polymeric surfactant composed of polyethylene oxide (PEO) and polypropylene oxide (PPO) in a block structure was employed as an interfacial dispersant to reduce the surface tension of silica and enhance its interfacial contact with TESPT, thereby improving the compatibility between silica and the rubber matrix. To further investigate the effect of hydrophilic–lipophilic balance (HLB) on the system performance, three types of block polyethers with HLB values of 8, 13, and 22 were selected and co-applied with TESPT to prepare NR-based nanocomposites via a one-step mixing method. Characterization by dynamic mechanical analysis (DMA), rubber process analyzer (RPA), and scanning electron microscopy (SEM) confirmed that the introduction of block polyether significantly promoted the dispersion of silica within the NR matrix and substantially enhanced both the static and dynamic mechanical properties as well as the processability of the composites. Experimental results further demonstrated that the HLB value of the block polyether has a notable influence on the overall performance of the materials.

## 2. Materials and Methods

### 2.1. Materials

Natural rubber (NR), SCR-WF, was produced by Yunnan Natural Rubber Industry Group (Kunming, China). Precipitated silica, VN3 GR (average particle size: 20–30 nm, BET specific surface area: 175 m^2^ g^−1^), was purchased from Evonik Industries (Essen, Germany). Phenyl alkylsulfonate (T-50), industrial grade, was purchased from Zhengzhou Yufeng Nanomaterials Co., Ltd. (Zhengzhou, China). Bis-[γ-(triethoxysilyl)propyl] tetrasulfide (TESPT), technical grade, was produced by Dongguan Kangjin New Material Co., Ltd. (Dongguan, China). N-cyclohexylbenzothiazole-2-sulphenamide and tetramethylthiuram disulfide, technical grade, were received from Shijiazhuang Aoho New Materials Co., Ltd. (Shijiazhuang, China). Sulfur, stearic acid, Poly(1,2-dihydro-2,2,4-trimethylquinoline), and N-isopropyl-N’-phenyl-p-phenylenediamine, industrial grade, were offered by Shijiazhuang Hongtai Chemical Technology Co., Ltd. (Shijiazhuang, China). Zinc oxide, technical grade, was purchased from Hebei Songzhi Chemical Science and Technology Co., Ltd. (Songzhi, China).

Block polyether P123 (HLB value 8, molecular weight 5750, abbreviated as BP8) was produced from Hai’an Petrochemical Plant (Hai’an, China). Block polyether P104 (HLB value of 13, molecular weight of 5900, abbreviated as BP13) was purchased from Jiasheng Chemical Co., Ltd. (Jiangyan, China). Block polyether F127 (HLB value 22, molecular weight 12600, abbreviated as BP22), was produced by BASF GmbH (Schwarzheide, Germany). The hydrophilic–lipophilic balance (HLB) of block polyether is an empirical index used to measure the relative proportion of hydrophilic and hydrophobic portions in nonionic surfactant molecules. It is commonly employed to predict the distribution behavior and surface activity of surfactants in different phases [29]. According to the cloud point method, a 10% aqueous solution of block polyether is placed in a large test tube with a liquid level of 50 mm. The solution is then stirred and slowly heated in a glycerin bath. When the solution becomes permanently turbid due to a decrease in transparency, the temperature inside the test tube corresponds to the cloud point of the block polyether. The HLB value is then calculated using the following equation [30,31]:

(1) HLB=0.098X+4.02

where X represents the cloud point of a 10% block polyether aqueous solution. By adjusting the lengths of the PPO and PEO segments in the block polyether, the HLB value and molecular weight can be controlled, allowing the production of products with varying grades [32].

### 2.2. Preparation of Nanosilica/Natural Rubber Composites

In this paper, silica-filled natural rubber composites were prepared using a two-step mixing process, and the detailed formulation of the NR composites is shown in Table 1. First, the NR raw rubber was placed in a two-roll mill (LRHI-200, Guangzhou Putong Experimental Analytical Instrument Co., Ltd., Guangzhou, China) at 80 °C with a roll speed ratio of 1.2 and moulded until it became semi-transparent, allowing for the smooth incorporation of the ingredients. The temperature of the internal mixer (LH60, Shanghai Kechuang Rubber and Plastics Machinery Co., Ltd., Shanghai, China) was then set to 100 °C, and the speed was set to 50 rpm. TESPT, block polyether, and silica were added sequentially, ensuring thorough mixing of all components. The temperature was then increased to 150 °C and mixed for 5 min to ensure the completion of the silanization reaction, after which the mixture was allowed to stand for several hours. In the second step, at 100 °C, stearic acid, zinc oxide, antioxidant, and T-50 were sequentially added to the mixed rubber in the internal mixer, with a total mixing time of 20 min. The mixture was then removed and allowed to cool naturally to room temperature. Finally, at room temperature, the accelerator and sulfur were added to the mixed rubber in the two-roll mill, and the mixture was passed at least six times to ensure uniform dispersion.

The optimal curing time (t_c90_) and scorch time (t_c10_) of the silica/NR composite were determined using a rotorless rheometer (M-3000A, Taiwan High Speed Rail Corporation, Taiwan, China). The mixed rubber was then cured at 150 °C, 10 MPa, and the optimal curing time (t_c90_) in a hydraulic press vulcanizer (XH-406BEW-50-300, Dongguan Xihua Testing Instruments Co., Ltd., Dongguan, China). Prior to performance testing, the composites were allowed to stand at room temperature for at least one day.

### 2.3. Characterization

The tensile strength and elongation at break of the vulcanized rubber were tested at 25 °C using a universal testing machine (MTSDW-20, Meters Testing Instruments Co., Ltd., Dongguan, China) according to ISO 37:2024 [33], with a stretching rate of 500 mm/min. Hardness was measured using a Shore A durometer (LX-A, Shanghai Gaozhi Precision Instruments Co., Ltd.) in accordance with ISO 48-4:2018 [34].

Strain scanning of the uncured silica/NR composite was performed using a rubber process analyzer (RPA) (RPA-8000, Taiwan High Speed Rail Corporation) at 60 °C and 1 Hz, with a scanning range of 0.1–400%.

A dynamic mechanical analyzer (DMA) (TA Q800, TA Instruments) was used to evaluate the dynamic mechanical properties of the vulcanized rubber composites. Rectangular specimens (10 mm × 5 mm × 2 mm) were subjected to temperature scanning at 10 Hz and 40 µm (amplitude) over a temperature range of −80 to 80 °C, with a heating rate of 3 °C/min.

The curing characteristics of the silica/NR composite were determined using a rotorless rheometer (M-3000A, Taiwan High Speed Rail Corporation) according to ISO 6502:2018 [35]. The torque–time curve was recorded at 150 °C.

The processing performance of the composite was tested using a torque rheometer (XSS-300, Shanghai Kechuang Rubber and Plastics Machinery Co., Ltd.), which records the variation in torque, energy, and other parameters over time during the entire mixing process.

The surface morphology of the tensile fracture section and filler distribution of the composite were observed using a scanning electron microscope (SEM) (TESCAN MIRA LMS, TESCAN Trading Co., Ltd., Singapore).

The abrasion resistance of the composites was then tested using an Akron abrasion machine (CZ-3005, Yangzhou Changzhe Testing Machinery Co., Ltd., Yangzhou, China) in accordance with ISO 23794:2023 [36]. Firstly, the sample was pre-ground for 600 revolutions, the rubber particles were brushed off, and the weight of the rubber wheel was measured to an accuracy of 0.001 g, recorded as $m_{1}$. Then, the pre-ground sample was further ground for 3416 revolutions, and the weight of the rubber wheel was measured again, recorded as $m_{2}$. The density (ρ) of the samples was tested according to ISO 2781:2018 [37]. The Akron abrasion volume V was calculated using the following equation:

(2) $$V=\frac{m_{1}-m_{2}}{\rho }$$

The rubber composite was cut into 10 × 10 × 2 mm^3^ pieces and weighed ($w_{1}$). Then, the rubber was immersed in 100 mL of toluene at room temperature and in the dark for 7 days. The swollen rubber was removed from the solvent, and the excess solvent was removed from its surface and weighed ($w_{2}$). The degree of swelling was determined using the following equation [38].

(3) $$\mathrm{Swelling}\;\mathrm{ratio}=\frac{w_{2}-w_{1}}{w_{1}}\times 100\%$$

The crosslink density ($V_{e}$) was then calculated using the Flory–Rehner equation [39]:

(4) $$V_{e}=-\frac{ln\left(1-V_{r}\right)+V_{r}+\chi_{1}V_{r}^{2}}{V_{1}\left(V_{r}^{1/3}-V_{r}/2\right)}$$

where $V_{1}$ is the molar volume of toluene (106.2 cm^3^ mol^−1^), $\chi_{1}$ is the rubber–toluene interaction parameter (0.393), and $V_{r}$ is the volume fraction of polymer in dissolved rubber in pure solvent and is calculated using the following equation [40]:

(5) $$V_{r}=\frac{w_{1}/\rho_{r}}{w_{1}/\rho_{r}+w_{2}/\rho_{s}}$$

where $\rho_{r}$ and $\rho_{s}$ are the densities of rubber and toluene (0.867 g cm^−3^), respectively.

## 3. Results and Discussion

### 3.1. Effect of HLB Value of Block Polyethers on Silica Dispersion in SiO_2_/NR Composites

**Figure 1:**
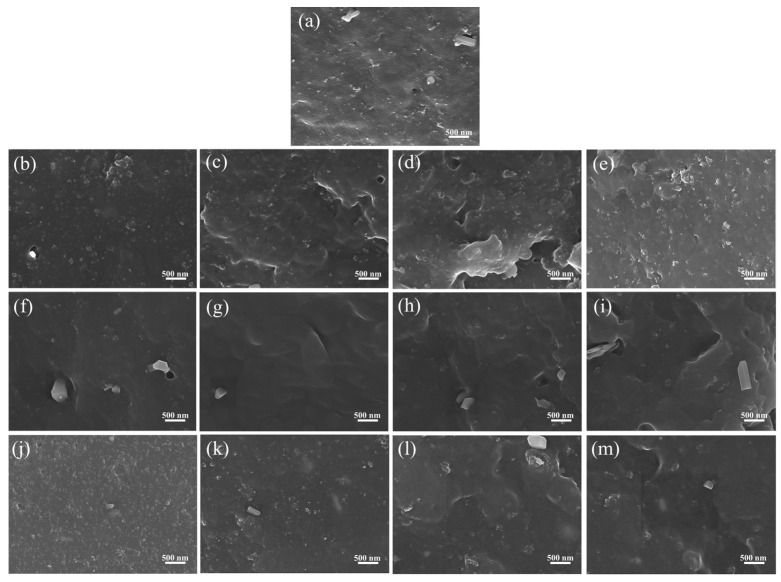
SEM images of tensile fracture surfaces of rubber composites: (**a**) Blank; (**b**) BP8-0.5; (**c**) BP8-1.0; (**d**) BP8-1.5; (**e**) BP8-2.0; (**f**) BP13-0.5; (**g**) BP13-1.0; (**h**) BP13-1.5; (**i**) BP13-2.0; (**j**) BP22 -0.5; (**k**) BP22-1.0; (**l**) BP22-1.5; (**m**) BP22-2.0.

Due to the tendency of silica to aggregate into clusters, and the silane coupling agents’ preference to undergo silylation reactions with the silicon hydroxyl groups on the surfaces of these aggregates, the modification efficiency is low. In this study, three block copolymers with HLB values of 8, 13, and 22 were used to investigate their effects on the properties of SiO_2_/NR composites at loadings of 0.5, 1.0, 1.5, and 2.0 phr. To study the dispersion of silica in the NR matrix, the microstructure of the tensile fracture section of the composite was observed, as shown in Figure 1. The block-like structures in the SEM images may represent aggregates of the filler, or dust and impurities, and therefore were not considered in the analysis.

**Figure 2:**
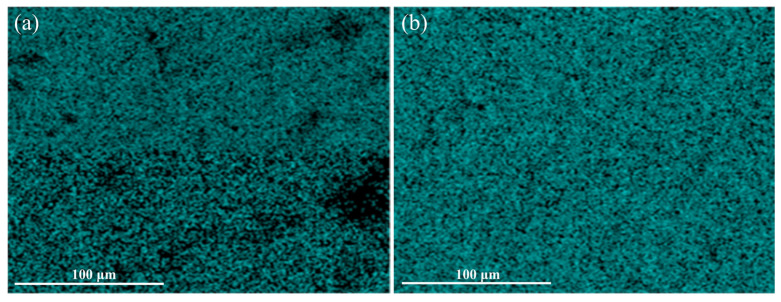
EDS mapping of Si element on the tensile fracture surfaces of SiO_2_/NR composites: (**a**) Blank; (**b**) BP13-1.0.

As shown in Figure 1a, the blank sample contains a significant amount of silica aggregates, indicating that simply adding TESPT for modification is insufficient to achieve effective filler dispersion. After adding the interface dispersing agent, the number of silica aggregates in the composite cross-section first decreases and then increases with the increase in the content of the three block copolymers. The composite material with 1 phr of block copolymer shows a more uniform filler distribution. This suggests that adding 1 phr of block copolymer is more effective in deconstructing and dispersing silica aggregates. The reason for this can be explained by the fact that the PEO chains on both sides of the block copolymer can form physical adsorption with the hydroxyl groups on the silica surface, while the PPO segment in the middle is compatible with the NR matrix, thus effectively promoting filler dispersion and the formation of interfacial interactions. Compared to the 1 phr sample, samples with more block copolymers exhibit poorer dispersion uniformity. This may be due to the plasticizing effect of excessive amounts of block copolymers in the system, which adversely affects the mixing process of the rubber compound [28]. Compared to the other two block copolymers, the composites containing BP13 exhibit smoother fracture surfaces, with the BP13-1.0 sample showing virtually no visible silica aggregates. The Si element distribution EDS maps of the Blank and BP13-1.0 samples in Figure 2 further confirm the uniform filler distribution in BP13-1.0. Among BP8, BP13, and BP22, BP13 possesses intermediate hydrophilicity, allowing it to effectively adsorb onto the surface of silica particles during mechanical shearing, thereby reducing aggregate formation and enhancing the silanization efficiency of TESPT. If the HLB value is too high, the dispersant has excessive affinity for silanol groups and tends to accumulate in the silica phase; conversely, a low HLB value favors its distribution in the rubber phase, which is insufficient to break down silica aggregates into finer particles. These findings demonstrate that the improved silica dispersion and the strong interfacial interactions between filler and rubber are the key factors by which block copolymers enhance the performance of silica/NR composites.

**Figure 3:**
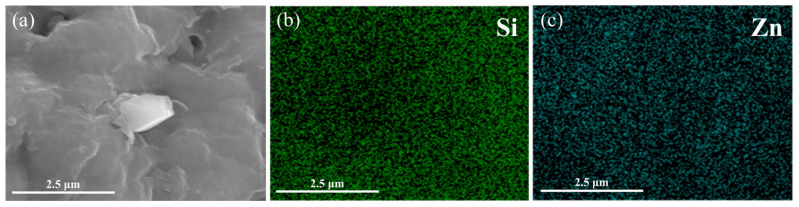
SEM and EDS images of the tensile fracture surface of the Blank sample: (**a**) SEM micrograph of the scanned area; (**b**) elemental mapping of Si on the fracture surface; (**c**) elemental mapping of Zn on the fracture surface.

It has been reported that zinc oxide may exist in rubber composites in the form of small crystals. To verify that the observed agglomerates in the samples are silica rather than zinc oxide, an EDS area scan was performed on the Blank sample, as shown in Figure 3. In Figure 3a, large particulates are observed on the fracture surface. However, Figure 3b,c show a relatively uniform distribution of Si and Zn elements within this region, indicating that the large particles in Figure 3a are not zinc oxide crystals, but are more likely to be impurities or dust. Furthermore, zinc oxide crystals typically exhibit well-defined crystalline morphologies, such as hexagonal columnar or plate-like structures, whereas silica agglomerates generally present as irregular particles or bulky aggregated structures. As shown in Figure 1, the agglomerates exhibit rough and irregular surfaces, further confirming that these aggregates are silica.

### 3.2. Influence of Block Polyethers on the Processing Properties of SiO_2_/NR Composites

**Figure 4:**
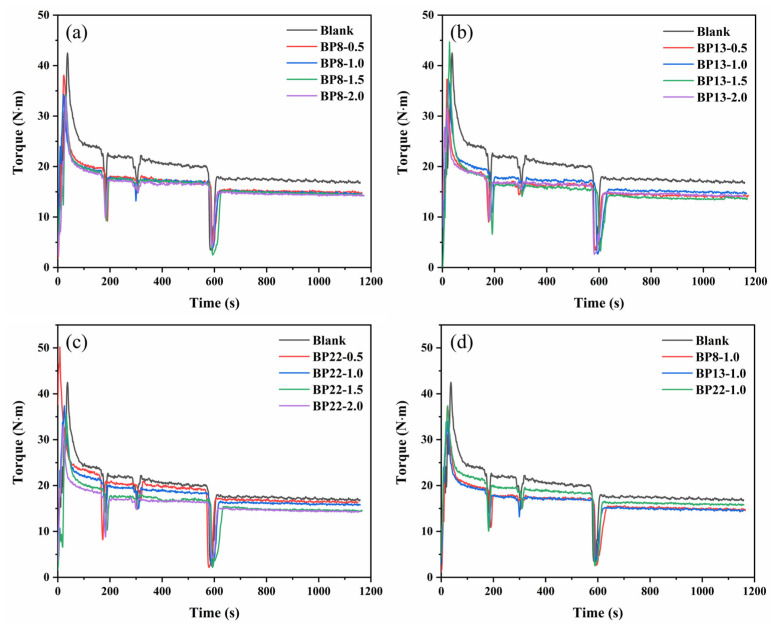
Effect of block polyethers on the mixing torque–time curve of NR compounds: (**a**) different amounts of BP8, (**b**) different amounts of BP13, (**c**) different amounts of BP22, and (**d**) different types of block polyethers with the same content.

To investigate the effect of block polyethers on the processability of the composites, a two-step mixing method was employed for the preparation of rubber composites. In the first mixing stage, 3 phr of silane coupling agent TESPT and varying amounts (0.5, 1.0, 1.5, and 2.0 parts) of block polyethers were added to the NR and SiO_2_ at 150 °C to achieve interfacial modification, resulting in masterbatch compounds. In the second mixing stage, processing additives such as zinc oxide and stearic acid were introduced into the compound to balance the performance. The influence of block polyethers with different dosages and hydrophilic–lipophilic balance (HLB) values on the mixing behavior of the NR compounds is shown in Figure 4.

As shown in Figure 4a, with an increasing mixing time and the addition of processing additives, the torque values of all samples gradually decreased. The final mixing torque eventually stabilized, indicating that the silica filler, once incorporated and dispersed, reached a particle size that could no longer be reduced under the given mixing conditions. Therefore, the mixing process was considered complete, as a optimistic level of filler dispersion was achieved [41]. Upon the introduction of block polyethers, Figure 4a,b reveal that compounds containing various types of block polyethers exhibited significantly lower mixing torque compared to the control sample without polyether, indicating that the addition of block polyethers can effectively enhance the processability of the composites. However, for BP8 and BP13, the torque values remained relatively constant regardless of the dosage. In contrast, Figure 4c shows that the torque of BP22-containing compounds continuously decreased with increasing dosage. This phenomenon may be attributed to the differing lubrication effects [42] and hydrophilic–lipophilic balance (HLB) characteristics of the dispersants. On one hand, BP22 possesses the highest polarity and molecular weight among the three dispersants. Its excessive polarity results in poor compatibility with the NR matrix, leading to migration toward the compound surface and predominantly exerting an external lubrication effect. On the other hand, due to the shorter hydrophilic segments and lower polarity of BP8 and BP13, these dispersants can more readily penetrate the gaps between rubber molecular chains, enhancing chain mobility and thereby acting mainly as internal lubricants. Furthermore, BP8 and BP13 contain more lipophilic groups in their structures and have lower molecular weights, which facilitates their incorporation into the rubber network. This results in a reduction in compound viscosity and allows them to effectively improve the processability of the composites even at low loadings. The BP22 structure contains a relatively higher proportion of hydrophilic groups, making it more likely to localize within the silica-rich phase. Its interaction with the rubber matrix is relatively limited, and due to its higher molecular weight, its loading has a more pronounced effect on the processability of the compound. Figure 4d analyses and compares the effects of block polyethers with different HLB values on the processing performance of the blended rubber with the content of all 1 phr. The torque order of the compounds is as follows: Blank > BP22-1.0 > BP8-1.0 ≈ BP13-1.0. This indicates that block polyethers with lower molecular weight and weaker hydrophilicity are more effective in reducing the mixing torque of the compound. In addition to the lubricating effect of the small molecules and the influence of the hydrophilic–lipophilic balance of properties, the improved dispersion of silica further improves the processability of the rubber, as shown in Figure 1.

### 3.3. Influence of Block Polyethers on the Vulcanization Characteristics of Silica/NR Composites

**Figure 5:**
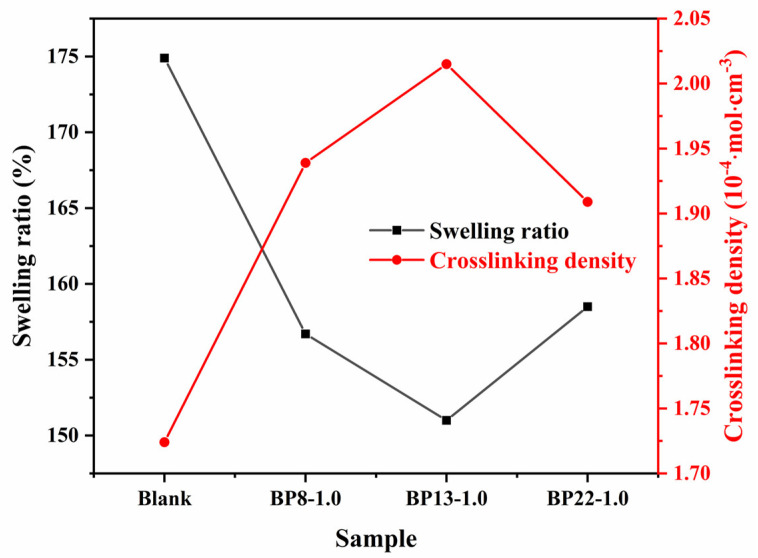
Crosslink density and swelling ratio of SiO_2_/NR adhesive.

To investigate vulcanization parameters such as curing time and temperature, as well as to analyze crosslink density, curing curves must be determined using a Moving Die Rheometer (MDR) prior to thermal vulcanization. The curing characteristics of silica/NR composites containing 0.5–2.0 phr of three block polyethers with different HLB values are summarized in Table 2. It can be observed that the addition of block polyethers slightly prolongs the optimum curing time (t_c90_) and also leads to a modest increase in scorch time (t_c10_). Among the three dispersants, the BP13 series exhibits a notably longer t_c10_. The increase in t_c10_ is closely related to the dispersion of silica. On one hand, block polyethers can effectively inhibit silica agglomeration, allowing for more efficient TESPT silanization and mitigating premature crosslinking. On the other hand, block polyethers can act as internal lubricants during mixing, reducing friction between the compound and the rotor, thereby minimizing shear heating and improving processing safety. Moreover, the torque difference (M_H_–M_L_) serves as an indicator of the crosslink density of the material [43]. It was observed that the composites containing block polyethers exhibited higher torque differences, indicating enhanced crosslinking between silica and the rubber matrix, as well as stronger filler–rubber interfacial interactions. The incorporation of block polyethers may also promote the formation of a filler network, thereby further increasing the crosslink density, as illustrated in Figure 5. Figure 5 compares the crosslink densities of composites containing 1 phr of different block polyethers. Among them, the BP13-1.0 sample exhibited the highest crosslink density, suggesting that at this particular HLB value, the compatibility between silica and natural rubber is significantly improved. This allows the block polyether to better localize at the filler–rubber interface, thereby enhancing interfacial interactions.

### 3.4. Effect of Block Polyethers on the Mechanical Properties of SiO_2_/NR Composites

The effects of three block polyethers with different HLB values at loadings of 0.5, 1.0, 1.5, and 2.0 phr on the mechanical properties of NR composites are summarized in Table 3. Upon the addition of block polyethers, both the tensile strength and modulus of the composites showed significant improvement, while hardness and abrasion resistance remained nearly unchanged. When the dosage of each block polyether reached 1.0 phr, the mechanical properties of the composites peaked. Further increases in block polyether content led to a decline in performance, which can be attributed to the plasticizing effect of excess block polyether that adversely affects material properties. Among the systems containing 1.0 phr of block polyether, BP13-1.0 demonstrated the most effective reinforcement, with a tensile strength of 28.9 MPa, a modulus at 300% elongation of 14.6 MPa, and an elongation at break of 523%. This indicates a substantial enhancement in strength without compromising ductility. Such behavior suggests that the segmental structure of the block polyether with HLB = 13 is optimal for silica dispersion. Due to its moderate hydrophilicity, BP13 tends to localize at the silica–rubber interface, reducing large particle agglomeration and facilitating the formation of more effective chemical and physical interfaces within the SiO_2_/NR composite. Generally, increases in tensile strength are accompanied by reductions in elongation at break and increases in hardness. However, upon incorporation of the block polyether, two mechanisms contribute to improved performance: on one hand, the block polyether forms a physical interface between silica and NR, allowing rubber chains to slide along the silica surface, thereby distributing stress and increasing elongation at break; on the other hand, it promotes the formation of a filler–rubber network. As a result of the reduced filler–filler network and enhanced filler–rubber interactions, the hardness of the SiO_2_/NR nanocomposites remains largely unchanged [44].

**Figure 6:**
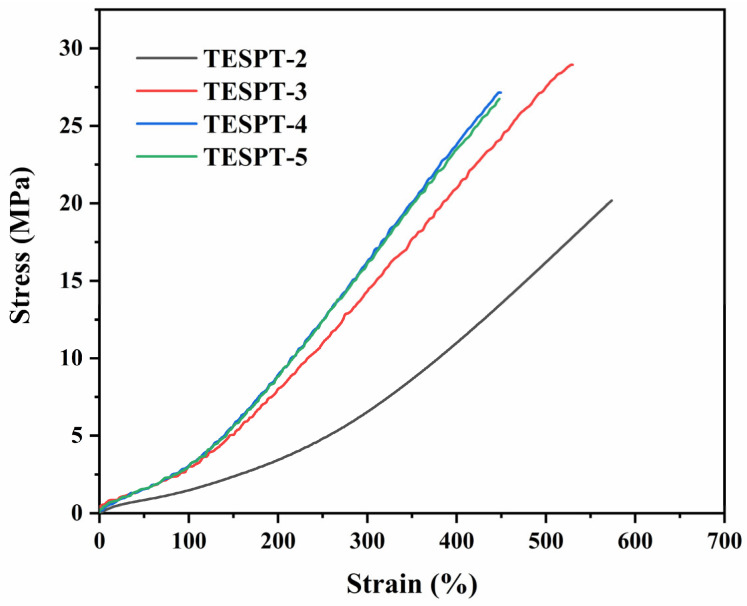
Stress–strain curves of composites with different TESPT contents.

According to the literature, the optimal dosage of TESPT is typically 8–10% of the silica content. As shown in Table 3, the BP13-1.0 composite exhibited the best mechanical performance, in which TESPT was used at 3 phr and the block polyether P104 at 1 phr. To investigate the effect of TESPT content on the composite system, while keeping the dosage of other additives unchanged and fixing the amount of block polyether P104 at 1 phr, composites were prepared with varying TESPT contents of 2, 3, 4, and 5 phr. These formulations were designated as TESPT-2, TESPT-3, TESPT-4, and TESPT-5, respectively. As illustrated in Figure 6, the TESPT-2 composite exhibited relatively poor mechanical properties, indicating poor compatibility between the filler and rubber matrix at low silane levels. This can be attributed to insufficient TESPT content, resulting in incomplete surface modification and residual silanol groups on silica particles. In contrast, when the TESPT dosage was increased to 4–5 phr, the differences in mechanical performance among the composites were marginal. While the modulus increased, a significant reduction in toughness was observed. This decline in ductility may be caused by an excessively high crosslink density or premature crosslinking during processing, both of which can compromise the final material performance. Therefore, within this formulation system, the optimal TESPT loading is determined to be 3 phr.

Based on the experimental results discussed above, it can be concluded that block polyethers contribute to the dispersion of silica and the enhancement of composite properties. In order to verify the interfacial role of block polyethers, the effect of block polyether P104 with HLB value of 13 on the properties of silica/NR composites is now investigated without the addition of TESPT. Composites with P104 dosages of 0, 3, 4, and 5 phr were designated as BP13-0, BP13-3, BP13-4, and BP13-5, respectively. Their mechanical properties are summarized in Table 4. As shown in the table, the BP13-0 sample, which contained no surface modifier, exhibited poor mechanical performance due to the presence of large silica agglomerates within the rubber matrix. These poorly dispersed aggregates significantly limit the reinforcing efficiency of the silica. Upon the addition of block polyether, the composites exhibited satisfactory performance even without silane coupling agent modification, achieving a tensile strength of up to 24.7 MPa and an elongation at break of 647%. This indicates that the block polyether can reduce silica agglomeration and decrease its polarity through physical adsorption, thereby improving the compatibility between the silica and rubber phases and enhancing interfacial interactions. However, excessive amounts of block polyether may lead to a plasticizing effect, which compromises the overall material properties. Although block polyether alone can impart favorable mechanical performance, molding defects were observed during processing. Moreover, modification via adsorption alone is insufficient to effectively eliminate the surface silanol groups of silica. Therefore, it is necessary to incorporate silane coupling agents in combination to better regulate the comprehensive performance of the composites.

**Figure 7:**
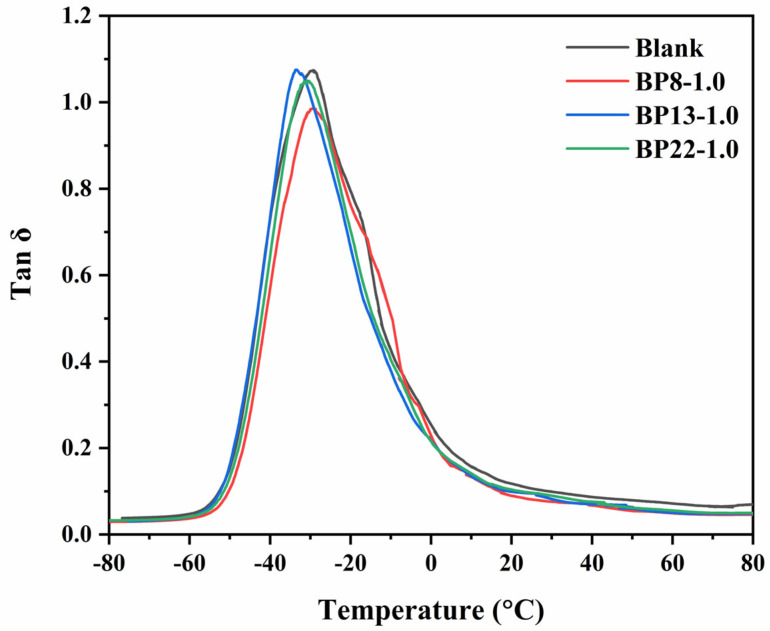
Temperature dependence of SiO_2_/NR composites.

As shown in Table 3, the composites exhibited superior mechanical properties when the dosage of each block polyether was fixed at 1 phr. However, the different HLB values of the block polyethers result in varying efficiencies in silica dispersion and silane coupling agent modification. To further investigate the influence of block segment distribution on the overall performance of the materials, three formulations, BP8-1.0, BP13-1.0, and BP22-1.0, were selected for viscoelastic analysis. Figure 7 presents the temperature-dependent dynamic mechanical behavior of the NR composites. During the temperature sweep test, a pronounced peak in the loss factor (tan δ) was observed in all samples. This peak corresponds to the glass transition process of the rubber composites, during which the molecular mobility of the rubber increases sharply, leading to significant hysteresis loss due to the lag in molecular motion [45]. Upon the addition of block polyethers, the glass transition temperature (T_g_) of the silica/NR composites exhibits minimal variation. However, the peak value of the loss factor (tan δ) at T_g_ shows a decreasing trend, with BP8-1.0 exhibiting the most significant reduction. This can be attributed to the improved interfacial interaction between NR and silica due to the incorporation of TESPT and block polyethers. Enhanced interfacial interactions can effectively suppress energy dissipation during molecular motion, leading to a lower tan δ peak at T_g_. On the other hand, improved silica dispersion disrupts the filler–filler network within the silica/NR composites, reducing the number of rubber macromolecular chains trapped within silica agglomerates. As a result, more free rubber chains are available to undergo segmental relaxation at T_g_, which tends to increase the peak tan δ value. These two effects coexist and compete with each other. In the BP13-1.0 system, the contributions from interfacial interactions and filler network disruption are balanced, resulting in a nearly unchanged tan δ peak. In contrast, for BP22-1.0 and BP8-1.0, the interfacial interaction dominates, leading to a decreased tan δ peak after modification. Moreover, compared to pure NR, the T_g_ of the modified composites increases. This is not only due to enhanced interfacial interactions between the rubber and the filler but also potentially due to the formation of a higher fraction of interfacial regions resulting from improved silica dispersion. These interfacial regions impose greater constraints on the mobility of rubber chains, resulting in reduced segmental motion and an elevated T_g_ [46,47].

### 3.5. Influence of Block Polyethers on the Payne Effect in Silica/NR Composites

**Figure 8:**
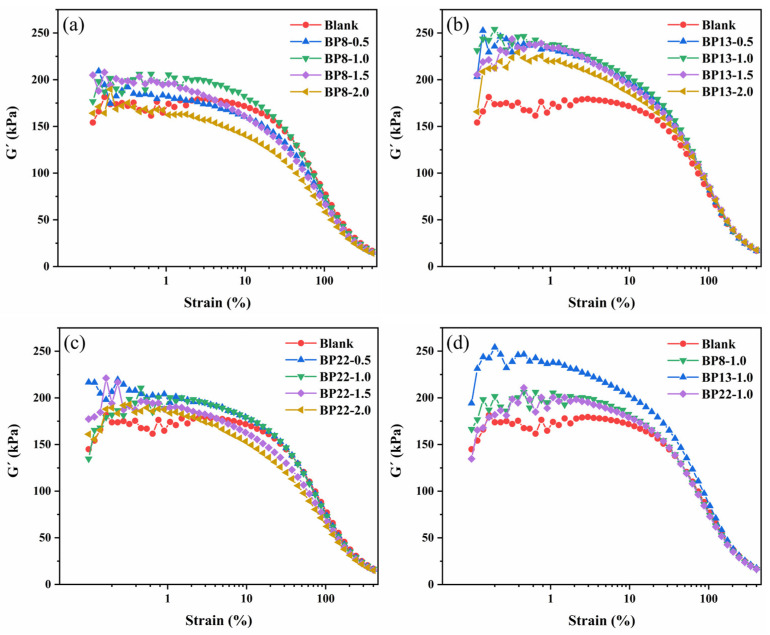
Strain amplitude dependence of silicon dioxide/NR unsulphide: (**a**) different amounts of BP8, (**b**) different amounts of BP13, (**c**) different amounts of BP22, and (**d**) block polyethers with different species with the same content.

Nanosilica/natural rubber (NR) composites exhibit the Payne effect, which is characterized by a pronounced decrease in storage modulus (G′) with increasing strain amplitude beyond the linear viscoelastic region [48]. Figure 8 illustrates the strain amplitude dependence of NR composites incorporating 0.5, 1.0, 1.5, and 2.0 phr of block polyethers. As shown in Figure 8a–c, under the same strain conditions, the systems with added block polyethers display higher G′ values, indicating an enhanced Payne effect. The differences in G′ among the various dosages of each block polyether are relatively small, but all show an upward trend, with the highest initial G′ observed when the block polyether dosage is 1 phr. In Figure 8d, a comparison among the three types of block polyethers reveals that BP13-1.0 exhibits the highest G′, while BP8-1.0 and BP22-1.0 show lower and comparable G′ values. This suggests that the chain segment distribution of the block polyether with an HLB value of 13 (BP13) is the most favorable for silica dispersion and for enhancing interfacial interactions between the filler and the NR matrix, as shown in the results of Figure 1 and Table 3. From the perspective of the chain segment distribution of the three block polyethers, the number of repeating units in the PPO segments remains nearly the same, while the difference lies in the number of repeating units in the PEO segments. The block polyether in the BP8-1.0 system has an HLB value of 8, making it more prone to aggregate within the rubber phase, with limited effect on reducing the surface energy of silica. In contrast, the block polyether in the BP22-1.0 system has an HLB value of 22, exhibiting strong hydrophilicity and tending to localize predominantly in the silica phase during mixing, but with insufficient distribution at the interface. The block polyether in the BP13-1.0 system has an HLB value of 13, which provides a balanced hydrophilic character. During compounding, BP13 can stably reside at the interface between silica and the NR matrix, effectively lowering the surface energy of silica, enhancing the silane coupling efficiency of TESPT, and improving interfacial interactions between silica and the rubber matrix. It also promotes filler mobility, enabling filler particles to act as new physical entanglement points [49,50]. As a result, the number of silica agglomerates is significantly reduced, allowing the previously encapsulated rubber chains to be released and participate in the glass transition process. This facilitates enhanced rubber–filler interfacial interaction, representing a deviation from the conventional understanding of filler network reinforcement.

## 4. Conclusions

In this paper, the role of the content and hydrophilic–lipophilic balance of three block polyethers with HLB values of 8, 13, and 22 on the properties of silica/natural rubber composites was investigated. The results revealed that block polyethers can effectively improve the dispersion of silica, as well as the processability and curing characteristics of the composites. This is attributed to the poly(ethylene oxide) (PEO) segments of the block polyethers, which exhibit good hydrophilicity and are compatible with silica, while the poly(propylene oxide) (PPO) segments, owing to their methyl groups, are more hydrophobic and thus compatible with the rubber matrix. These dual compatibilities enable block polyethers to act as coupling bridges at the silica–rubber interface. Among the tested polyethers, the block polyether with an HLB value of 13 demonstrated the most favorable performance in the composite system. At a loading level of 1 phr, the composite exhibited higher crosslink density and a more pronounced Payne effect, indicating enhanced formation of the filler–rubber network. Consequently, the silica/NR composites showed improved tensile strength and modulus, along with reduced loss tangent (tan δ). Furthermore, the improved dispersion of silica within the NR matrix contributed to better mechanical properties and enhanced processability of the composites.

## Figures and Tables

**Table 1:** Formulation of silica/NR composites containing different block polyethers.

Ingredients (phr ^a^)	NR	Silica	TESPT	Block Polyether P104	Block Polyether P123	Block Polyether F127	Other Additives ^b^
Blank	100	50	3	0	0	0	17.8
BP8-0.5	100	50	3	0	0.5	0	17.8
BP8-1.0	100	50	3	0	1	0	17.8
BP8-1.5	100	50	3	0	1.5	0	17.8
BP8-2.0	100	50	3	0	2	0	17.8
BP13-0.5	100	50	3	0.5	0	0	17.8
BP13-1.0	100	50	3	1	0	0	17.8
BP13-1.5	100	50	3	1.5	0	0	17.8
BP13-2.0	100	50	3	2	0	0	17.8
BP22-0.5	100	50	3	0	0	0.5	17.8
BP22-1.0	100	50	3	0	0	1	17.8
BP22-1.5	100	50	3	0	0	1.5	17.8
BP22-2.0	100	50	3	0	0	2	17.8

^a^ Parts per hundred of rubber. ^b^ Zinc oxide 5; stearic acid 2; N-cyclohexylbenzothiazole-2-sulphenamide 1.5; Tetramethylthiuram disulfide 0.3; Poly(1,2-dihydro-2,2,4-trimethylquinoline) 1.5; N-isopropyl-N’-phenyl-p-phenylenediamine 1; sulfur 1.5; T-50, 5.

**Table 2:** Vulcanization characteristics of silica/NR compounds.

Properties	t_c10_ (min:s)	t_c90_ (min:s)	M_L_ (dNm)	M_H_ (dNm)	M_H_-M_L_ (dNm)
Blank	3:15	5:34	1.44	12.76	11.32
BP8-0.5	3:38	5:54	1.42	13.81	12.39
BP8-1.0	3:56	5:56	1.64	16.40	14.76
BP8-1.5	3:52	5:59	1.74	17.29	15.55
BP8-2.0	3:51	6:02	1.65	16.87	15.22
BP13-0.5	4:22	6:46	1.18	14.22	13.04
BP13-1.0	3:53	5:58	1.57	16.29	14.72
BP13-1.5	4:02	6:03	1.23	16.23	15.00
BP13-2.0	4:06	5:56	1.09	15.55	14.46
BP22-0.5	3:49	6:06	1.26	14.87	13.61
BP22-1.0	3:43	5:43	1.64	16.18	15.17
BP22-1.5	4.01	6:02	1.07	15.33	14.26
BP22-2.0	3:38	5:33	1.40	17.66	16.26

**Table 3:** Mechanical properties of SiO_2_/NR composites.

Properties	Tensile Strength (MPa)	Elongation at Break (%)	Modulus at 100% Elongation (MPa)	Modulus at 300% Elongation (MPa)	Shore A Hardness	Abrasion Loss (cm^3^)
Blank	25.3 ± 0.1	494 ± 15	2.6 ± 0.1	12.6 ± 0.4	60 ± 1	0.57 ± 0.02
BP8-0.5	26.5 ± 0.2	529 ± 30	2.2 ± 0.3	12.1 ± 1.3	62 ± 1	0.56 ± 0.03
BP8-1.0	27.6 ± 0.5	516 ± 7	2.6 ± 0.1	13.3 ± 0.8	60 ± 1	0.55 ± 0.01
BP8-1.5	25.2 ± 0.5	512 ± 28	2.4 ± 0.2	12.5 ± 0.9	60 ± 1	0.54 ± 0.02
BP8-2.0	23.9 ± 0.4	479 ± 16	2.4 ± 0.1	12.1 ± 0.3	58 ± 1	0.52 ± 0.02
BP13-0.5	27.8 ± 0.3	528 ± 34	2.6 ± 0.3	13.6 ± 1.6	59 ± 1	0.50 ± 0.01
BP13-1.0	28. 9 ± 0.1	523 ± 18	3.0 ± 0.1	14.6 ± 0.7	61 ± 1	0.50 ± 0.01
BP13-1.5	26.5 ± 0.4	501 ± 20	2.6 ± 0.2	13.7 ± 1.0	61 ± 1	0.50 ± 0.02
BP13-2.0	26.4 ± 0.1	481 ± 5	3.4 ± 0.1	14.7 ± 0.4	61 ± 1	0.50 ± 0.03
BP22-0.5	27.4 ± 0.4	521 ± 8	2.6 ± 0.1	13.5 ± 0.1	61 ± 1	0.51 ± 0.01
BP22-1.0	27.3 ± 0.3	494 ± 12	2.7 ± 0.2	14.0 ± 0.6	60 ± 1	0.50 ± 0.02
BP22-1.5	25.3 ± 0.6	460 ± 12	2.3 ± 0.1	11.8 ± 0.1	58 ± 1	0.51 ± 0.02
BP22-2.0	24.7 ± 0.3	494 ± 5	2.4 ± 0.1	11.7 ± 0.3	57 ± 1	0.52 ± 0.01

**Table 4:** Effect of block polyethers on mechanical properties of silica/NR composites (without TESPT modification).

Properties	Tensile Strength (MPa)	Elongation at Break (%)	Modulus at 100% Elongation (MPa)	Modulus at 300% Elongation (MPa)	Shore A Hardness
BP13-0	6.2 ± 0.3	622 ± 41	0.5 ± 0.1	1.4 ± 0.2	34 ± 1
BP13-3	24.7 ± 0.4	647 ± 14	1.4 ± 0.3	6.1 ± 0.1	62 ± 1
BP13-4	24.3 ± 0.2	584 ± 7	2.0 ± 0.1	7.7 ± 0.2	61 ± 1
BP13-5	22.4 ± 0.3	582 ± 12	1.9 ± 0.1	7.0 ± 0.1	61 ± 1

## Data Availability

Data are contained within the article.
